# Specific Recognition of Breast Cancer Cells In Vitro Using Near Infrared-Emitting Long-Persistence Luminescent Zn_3_Ga_2_Ge_2_O_10_:Cr^3+^ Nanoprobes

**DOI:** 10.1007/s40820-014-0026-0

**Published:** 2014-12-24

**Authors:** Jinlei Li, Junpeng Shi, Jiangshan Shen, Huizi Man, Mingxi Wang, Hongwu Zhang

**Affiliations:** 1 School of Biomedical Sciences, Huaqiao University and Engineering Research Center of Molecular Medicine, Ministry of Education, Xiamen, 361021 People’s Republic of China; 2 Institute of Urban Environment, Chinese Academy of Science, Xiamen, 361021 People’s Republic of China

**Keywords:** Nanoprobes, Long afterglow, Near-infrared luminescence, Target imaging

## Abstract

In this paper, near-infrared emitting long-persistence luminescent Zn_3_Ga_2_Ge_2_O_10_:Cr^3+^ (ZGG) nanoparticles with diameters of 30–100 nm and bright luminescence were prepared by a sol–gel synthesis method. After the surface amination, the nanoparticles were further bioconjugated with breast cancer-specific monoclonal antibody (anti-EpCAM) to form ZGG-EpCAM nanoprobes which can specifically target breast cancer cell lines (MCF7) in vitro. The results of in vitro images show that the luminescence signals from the cells treated with ZGG-EpCAM nanoprobes are stronger than those from cells treated with ZGG-unconjugated antibody, indicating that the prepared ZGG-EpCAM nanoprobes possessed excellent specific recognition capability. Furthermore, due to their long afterglow properties, the imaging could persist more than 1 h. Therefore, these nanoprobes could not only provide a high specificity detection method for cancer cells but also realize the long-time monitoring. Developed near-infrared emitting long-persistence luminescent nanoprobes will be expected to find new perspectives for cell therapy research and diagnosis applications.

## Introduction

Optical imaging techniques have excellent potential to help us further understand biological processes at the molecular level and provide useful tools for sensitive cancer diagnosis [1]. Biological optical imaging greatly relies on the use of sensitive and stable optical probes. To date, organic dyes [2, 3], quantum dots [4–6], metal nanoparticles [7–9], and upconverting nanoparticles [10] are the most commonly used optical labels probes, but they also have intrinsic limitations. Organic dyes suffer from a short observation time, partly due to photobleaching, poor stability under physiological conditions, and limited Stokes shift. Quantum dots display good photostability, size-dependent emissions, and high quantum yield, while they still require constant excitation, which is responsible for undesirable autofluorescence and enhanced background signal that reduce the sensibility of the assay [11, 12]. Such inherent disadvantages would affect their application for imaging in vitro or in vivo.

In order to overcome some of inherent problems encountered with optical imaging probes [13], persistent luminescence nanoparticles have been developed. It comes down to a particular optical phenomenon in which the excitation energy can be reserved by the material and then slowly released by a photonic emission, which can be persistent luminescence for some hours [14, 15]. After long-persistence luminescent nanoparticles are excited in vitro, real-time in vivo and vitro imaging can be achieved without needing any external illumination source. Therefore, the signal-to-noise ratio (SNR) can be significantly improved by eliminating the background noise originating from in situ excitation [16, 17]. Moreover, the long-persistent luminescence of near-infrared (NIR) emitting long-persistence luminescent nanoparticles (NLPLNPs) (the afterglow wavelength varies from 650–900 nm) pertain to the tissue transparency window, where light attenuation is largely due to scattering rather than absorption, which is advantageous for long-term in vivo and vitro imaging with deep penetration and a high SNR [18]. So far, large numbers of NIR persistent luminescence nanoprobes have been applied for imaging in vitro and in vivo. For example, they have been used to diagnose tumors and realize long-time monitoring of tumors [19] and have also been utilized to develop novel monodisperse NIR emitting long-persistence luminescent nanoparticles with a porous structure to realize not only tumor diagnosis but also the drug delivery [20]. Recently, non-toxic NLPLNPs with a narrow size distribution were utilized to realize tumor-targeting and efficient cell tagging to track the biological fate of cells in vivo in healthy tumor [21]. Although the NLPLNPs have displayed excellent advantages for long-time in vivo imaging with a high SNR, few researches focused on long-time cell imaging which is one of these important parts in bioimaging. In particular, up to now, NLPLNPs still no effectively realize specific recognition of cancer cells in vitro due to lack of suitable surface functional groups. Therefore, developing NLPLNPs with specific recognition agent is important for the long-time cell imaging.

In this paper, we utilized NIR emitting long-persistence luminescent Zn_3_Ga_2_Ge_2_O_10_:Cr^3+^ (ZGG) nanoparticles as targeted optical probes for labeling human breast cancer cells. The antibody anti-EpCAM, where corresponding antigen receptors are known to be overexpressed in both primary and metastatic breast cancer [22], were utilized as specific recognition agent to functionalize ZGG surface. ZGG-EpCAM can specifically recognize the long-term monitored breast cancer cells (MCF7) without persistent activation, which can obviously improve the SNR. With confocal microscopy and IVIS Lumina II imaging system, we demonstrate receptor-mediated recognition ZGG-EpCAM bioconjugates to breast cancer cells.

## Experimental Sections

### Reagents

Breast cancer cell lines MCF7 were obtained from the Cell Resource Center, Shanghai Institutes for Biological Sciences (SIBS, China). RPMI 1640 medium and fetal bovine serum (FBS) were purchased from Life Technologies (Gibco, USA). Phosphate buffered saline (PBS) of pH 7.2 and 0.25 % trypsin solution were obtained from the company (Thermo Scientific, USA). 1-Ethyl-3-[3-dimethylaminopropyl] carbodiimide hydrochloride (EDC), *N*-hydroxysuccinimide (NHS), 3-(4,5-dimethyl-2-thiazolyl)-2,5-diphenyl-2*H*-tetrazolium bromide (MTT), dimethyl sulphoxide (DMSO), and tetraethyl orthosilicate (TEOS), (3-aminopropyl) triethoxysilane (APTES) were purchased from Sigma-Aldrich (Sigma, USA). Anti-EpCAM monoclonal antibody (C-10) was obtained from Santa Cruz Biotechnology Corporation (Santa, USA). Ga(NO_3_)_3_·9H_2_O, GeO_2_, Zn(NO_3_)_3_·9H_2_O, and Cr(NO_3_)_3_·9H_2_O were purchased from Sigma-Aldrich (Shanghai, China). Ammonia 28 % solution, citric acid monohydrate, and ethanol absolute were obtained from Sinopharm Chemical Reagent (Shanghai, China).

### Synthesis of Long Afterglow NIR ZGG Nanoparticles

ZGG nanoparticles were synthesized by a sol–gel method. Zn(NO_3_)_2_·6H_2_O, Ga(NO_3_)_3_·6H_2_O, and Cr(NO_3_)_3_·9H_2_O were, respectively, dissolved in ultrapure water. The above four solutions were mixed together with the molar ratio of Zn^2+^:Ga^3+^:Ce^4+^:Cr^3+^ = 600:400:400:1. Then 2 g of citric acid was added and continuously stirred for 30 min. Subsequently, the solution was kept at 70 °C for 6 h under continuously stirring to form a highly viscous gel, and then the viscous gel was dried at 120 °C for 8 h. Finally, the precursor gel was sintered at the temperature of 800 °C for 2 h in muffle furnace.

### Surface Modifications of Long Afterglow NIR ZGG Nanoparticles

The surface modification of ZGG with APTES to get SiO_2_ and NH_2_ layers was performed according to previous report [19, 20]. In detail, the ZGG nanoparticles were firstly hydroxylated by basic wet grinding of the solid (40 mg) for 30 min with a mortar and pestle in 5 mM NaOH solution. The hydroxylation was performed overnight by dispersing the ground powder in 80 mL NaOH solution. After standing for 6 h, the supernatant was collected by centrifugal separation at 3,000 rpm for 10 min, and the sediment was dried at 80 °C for 8 h. The hydroxyl-modified ZGG particles were obtained. The 40 mg of hydroxyl-ZGG particles was dispersed into the mixture of 66.7 mL ethyl ethanol, 13.3 mL ultrapure water, and 5 mL concentrated aqueous ammonia (28 wt%) and ultrasonically treated for 50 min. The obtained suspension was put into 10 °C water bath with continuous magnetic stirring for 30 min, and then 0.1 % (by volume) TEOS alcoholic solution was dropwise added. After that another 0.1 % (by volume) alcoholic solution was added and stirred for 10 h. Finally, the ZGG-NH_2_ product was obtained by centrifugally separation at 10,000 rpm and washed with absolute ethyl ethanol for three times and dried in vacuum at 60 °C for 5 h. Functionalization of ZGG-NH2 conjugated with an anti-EpCAM antibody. 10 mg of ZGG-NH_2_ was immersed in 10 mL of PBS and ultrasonically treated for 30 min to form the ZGG-NH_2_ solution. 20 μL of anti-EpCAM (200 μg mL^−1^) was diluted by 10 mL of PBS. 15 mg of EDC and 10 mg of NHS powder were added into the diluted solution, and then the carboxyl anti-EpCAM solution was activated. The above two solutions were mixed thoroughly and then centrifuged and washed with PBS for three times to obtain the conjugation of ZGG-EpCAM.

### Characterization

X-ray powder diffractions (XRD) were performed on Panalytical X’pert PRO diffractometer equipped with Cu Kα radiation (*λ* = 1.5418 Å) at room temperature. Transmission electron microscopy (TEM) experiment was conducted on HITACHI H-7650 system. Hydrodynamic size distribution and zeta potential were measured by dynamic light scattering method (DLS) using a Malvern Zeta-sizer 3000 HS. The infrared spectra were obtained on a Fourier transform infrared spectroscopy with KBr pellet techniques (FTIR, Nicolet AVATAR, FTIR360). Optical imaging in cancer cells was achieved in an in vivo imaging system (Caliper Life sciences, IVIS lumina II) and Confocal Laser Scanning Microscopy (ZEISS, LSM710). The spectral properties (excitation and emission spectra, decay curves, long afterglow emission, and excitation spectra) of the ZGG nanoparticles were measured using an Instrument FS920 spectrometer fluorescence spectrometer.

### Cytotoxicity Test In Vitro

Cytotoxicity was measured by MTT assay. Briefly, MCF7 were seeded in 96-well plates (CORNING, USA) for 12 h. Then, after the old medium was removed, fresh medium containing ZGG-NH_2_ nanoprobes with different concentrations was added and incubated for 24 h at CO_2_ incubator (Thermo heracell 150i, USA). Subsequently, MTT was added and incubated for 4 h at CO_2_ incubator. The medium was removed, and DMSO was added to dissolve the formazan for 20 min. Finally, the absorbance at 490 nm was measured using a microplate reader (Molecular Devices, Sunnyvale, CA, USA), with a reference at 650 nm.

### In Vitro Imaging of Cells

For observing in vitro imaging of ZGG-EpCAM, MCF7 cells were cultured in RMPI-1640 medium containing 10 % FBS, 1 % penicillin, and 1 % amphotericin B. An appropriate number of MCF7 cells were firstly seeded in 35-mm culture dishes for 12 h before the treatment day. And then the seeded cells in serum-supplemented media were treated with 200 μL ZGG-EpCAM nanoprobes (50 μg mL^−1^) for 2 h at 37 °C. After 2 h, the cells were washed several times with PBS and directly imaged by a confocal microscope. In order to confirm that anti-EpCAM of modified ZGG-NH_2_ could selectively bound with EpCAM protein of the cells, MCF7 cells were incubated with 200 μL unmodified ZGG-NH_2_ (50 μg mL^−1^) and 5 μL anti-EpCAM (1 mg mL^−1^) blocking solution before addition of 200 μL ZGG-EpCAM nanoprobes (50 μg mL^−1^) as control. Confocal microscopy images were obtained using a fluorescence imaging system with laser excitation at 405 nm. The in vitro images of these two samples were obtained using IVIS Lumina II imaging system with UV lamp laser excitation for 2 min at 254 nm. All images were taken at the same condition. The pictures were taken per every 5 min, and the detecting time is 20 min.

## Results and Discussion

**Scheme 1:**
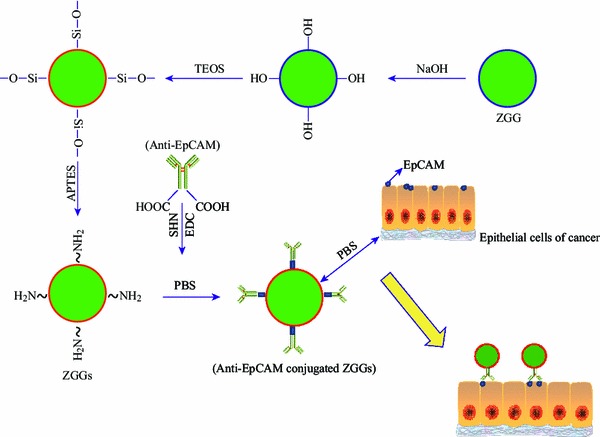
Schematic illustration of the process of functionalization of Zn_3_Ga_2_Ge_2_O_10_:Cr^3+^ (ZGG) and conjugated with anti-EpCAM finally to specially identify cells of MCF7 cell lines

The design and preparation process of long afterglow luminescent ZGG-EpCAM nanoprobe for cellular targeting and imaging was illustrated in Scheme 1. Firstly, the functionalization of ZGG nanoparticles could enhance the amount of –OH groups in NaOH solution. Subsequently, after the reaction with TEOS and APTES, a thin layer of SiO_2_ and NH_2_ groups were anchored on the ZGG-OH nanoparticle surface. Finally, in the presence of EDC and NHS, anti-EpCAM could react with –NH_2_ groups on the ZGG-NH_2_ nanoparticle surface to acquire recognition site and then the ZGG-EpCAM nanoprobe was obtained. It will be used to identify the cancer cells which could overexpress EpCAM antigen.

**Fig. 1:**
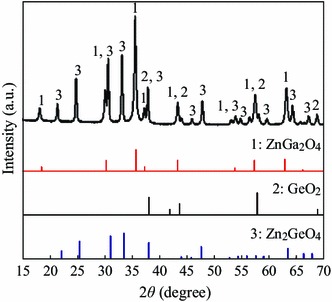
XRD profile of ZGG nanoparticles

**Fig. 2:**
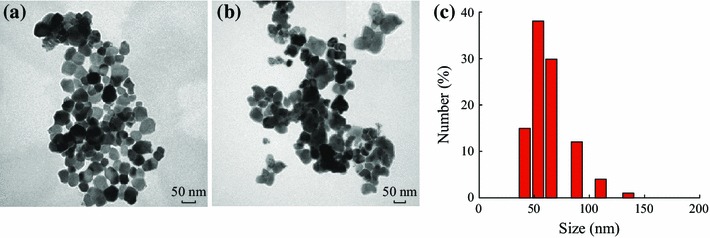
**a** TEM image of sol–gel method synthesized ZGG nanoparticles (*scale bar* is 50 nm); **b** TEM image of ZGG-NH_2_ (*scale bar* is 50 nm); **c** Dynamic light scattering hydrated diameter pattern of ZGG-EpCAM

The ZGG nanoparticles were systematically characterized by powder XRD. The XRD result of the ZGG nanoparticles is shown in Fig. 1. It shows that the phase of ZGG is in good agreement with reference data for the cubic phase of ZnGa_2_O_4_ (JCPDS card no. 86-0415), rhombohedral structure of Zn_2_GeO_4_ (JCPDS card no. 011-0687), and hexagonal structure of GeO_2_ (JCPDS card no. 34-1089), suggestive of the presence of a mixture of ZnGa_2_O_4_, Zn_2_GeO_4,_ and GeO_2_ in ZGG. Transmission electron microscopy illustrates that ZGG nanoparticles possess diameters ranging from 30 to 100 nm and some nonspherical shapes with different aspect ratios (Fig. 2a). After capped with a layer of SiO_2_ and NH_2_ groups, the diameter of ZGG-NH_2_ did not obviously changed compared with ZGG as shown in Fig. 2b. DLS analysis manifested the effective hydrodynamic size and size distributions of ZGG-EpCAM nanoparticles suspensions in ultrapure water. It demonstrated that the nanoparticles suspensions had effective hydrodynamic diameters ranging from 35 to 140 nm, which mainly distributed in the range of 50–70 nm (Fig. 2c). It is well known that narrow size distribution of nanoparticle is important for bioimaging. The diameter which is less of 100 nm is fit for application in vitro imaging.

**Fig. 3:**
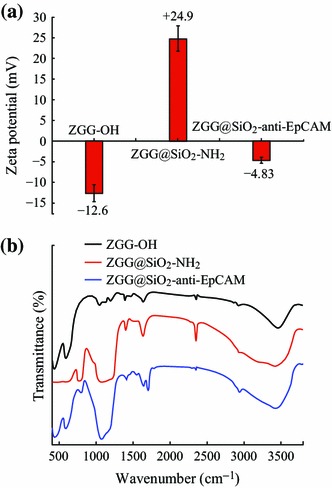
**a** FT-IR spectra of ZGG-OH, ZGG-NH_2_, and ZGG-anti-EpCAM; **b** Zeta potential of ZGG-OH, ZGG-NH_2_, and ZGG-EpCAM at neutral pH

In order to improve the biocompatibility, solubility, and biological specificity of ZGG, anti-EpCAM monoclonal antibody was grafted onto the surface of ZGG. The epithelial cell adhesion molecule (EpCAM) is a monomeric membrane glycoprotein that can be expressed in most normal human epithelium and overexpressed in most carcinomas. It has become a potential target for the visualization and therapy of human solid tumors [23–25]. The surface zeta potentials of the nanoparticles during different surface modification processes were used to monitor the change of surface functional group, as shown in Fig. 3a. After the reaction with NaOH, zeta potential of the sample became negative (−12.6 mV) which proved that hydroxyl groups were modified on the ZGG surface. However, the samples further treated with APTES gave the positive surface potential (+24.9 mV) as a result of the amino groups at the surface. Furthermore, the anti-EpCAM layer was grafted on the nanoparticles, zeta potential switched from positive to nearly values (−4.83 mV). In order to confirm the modification process, the FTIR spectra analysis was utilized, as shown in Fig. 3b. The strong FTIR band of ZGG-OH at 3,415 cm^−1^ shows the successful hydroxylation of the ZGG surface. The FTIR peaks of ZGG-NH_2_ at 3270, 1670, and 1051 cm^−1^ could be attributed to the stretching vibration of N–H and –Si–O–Si– bonds, which indicate the existence of amino groups on the surface of ZGG via modification of APTES. The peak of ZGG-EpCAM at 1,725 cm^−1^ could be attributed to the stretching vibration of C=O bonds, indicative of the success of surface modification with anti-EpCAM monoclonal antibody. All the results suggest that the anti-EpCAM had been successfully conjugated on the surface of ZGG-NH_2_ to form specific recognition ZGG-EpCAM nanoprobes.

**Fig. 4:**
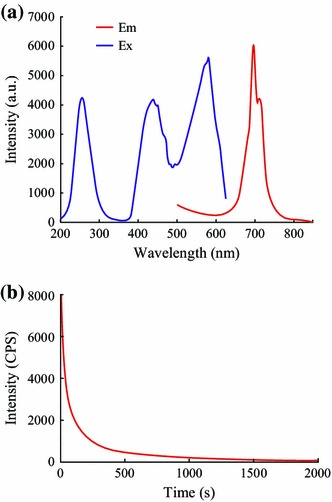
**a** Excitation (*λ*_em_ = 698 nm) and emission (*λ*_ex_ = 264, 426, and 575 nm) spectra of ZGG; **b** NIR long afterglow decay curve of ZGG-NH_2_ monitored at 698 nm after irradiated by 254 nm light for 5 min. The *inset* showed long afterglow emission of ZGG-NH_2_ nanoprobes recorded at 5 min after stopping irradiation

The emission spectrum of ZGG-EpCAM nanoprobes at room temperature (Fig. 4a) consists of a narrow-band emission peaked at 698 nm under excitation at 254 nm. This NIR emission was characteristic of Cr^3+^ ions and could be attributed to the spin-forbidden ^2^E → ^4^A_2_ transition. The excitation spectrum monitored at 698 nm consists of three main excitation bands originating from the inner transitions of Cr^3+^ located at 264, 426, and 575 nm, ascribed to the Cr^3+^, ^4^A_2_ → ^4^T_1_(te^2^) ^4^A_2_ → ^4^T_1_(t^2^e), and ^4^A_2_ → ^4^T_2_(t^2^e) transitions, respectively [26]. Furthermore, ZGG-EpCAM nanoprobes show excellent long afterglow NIR properties. The long afterglow intensity monitored at 698 nm after irradiation by 254 nm UV light for 5 min which was found to decrease in the first several minutes, and then slowly decayed. After 3 h of persistent emission, the persistent luminescence intensity was still kept (Fig. 4b).

**Fig. 5:**
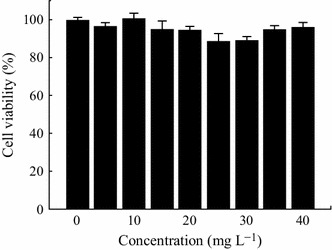
MTT assay on ZGG-NH_2_ nanoprobes treated MCF7 cells, where nanoprobes concentrations were 5, 10, 20, 30, and 40 mg L^−1^ and incubation time was 24 h

It is well known that low toxicity of nanoprobes is critical for in vivo or in vitro studies [19–21]. Herein, the cytotoxicity of ZGG-NH_2_ to MCF7 cells was examined by employing MTT method. As shown in Fig. 5, there are no obvious changes in the viability of MCF7 cells with the increasing concentrations of ZGG-NH_2_ after 24 h of incubation. Even though the concentration of ZGG-NH_2_ reached up as high as 40 mg L^−1^, the viability of MCF7 cells could keep in 98.9 %. This result shows that the ZGG-NH_2_ has no obvious cytotoxicity and is suitable for in vitro imaging.

**Fig. 6:**
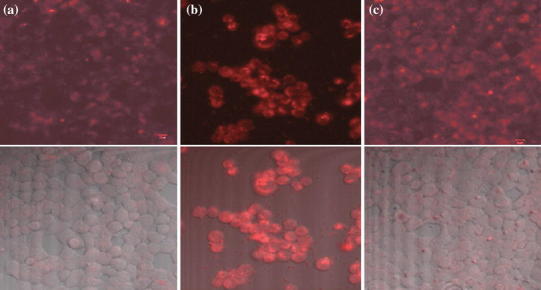
Confocal microscopic images of **a** MCF7 cells treated with ZGG-NH_2_; **b** MCF7 cells treated with ZGG-EpCAM; **c** MCF7 cells treated with ZGG-EpCAM after using blocking solution anti-EpCAM. In all cases, the *red* represents the emission from ZGG. (Top images were luminescence images and bottom images were white light images overlaid on top luminescence images). (Color figure online)

 In the in vitro imaging study, the ZGG-NH_2_ and ZGG-EpCAM were utilized to label human breast cancer cells (MCF7), as shown in Fig. 6a and b. Weak luminescence signal could be observed in MCF7 cells treated with ZGG-NH_2_ solution, while strong luminescence signal could be clearly observed in the case of MCF7 cells treated with ZGG-EpCAM (Fig. 6b), which could confirm the specific recognition of ZGG-EpCAM to MCF7 cells. In addition, when the blocking solution (add anti-EpCAM before adding ZGG-EpCAM) was used to eliminate the ability of anti-EpCAM to recognize human breast cancer cells (MCF7), weaker luminescence signal was observed in MCF7 cells (as shown in Fig. 6c), which indicated that the targeting capability of ZGG-EpCAM to breast cancer cells can be ascribed to the anti-EpCAM conjugated on surface. These results support that the engineered ZGG-EpCAM could specially recognize EpCAM overexpressing cells. Therefore, the engineered ZGG-EpCAM bioconjugates could serve as a potential biocompatible targeted nanoprobe to specifically diagnose human breast cancer cells.

**Fig. 7:**
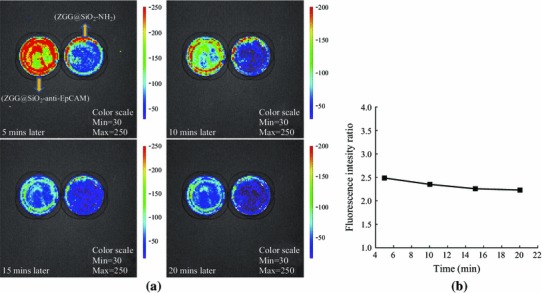
In vitro imaging of **a** ZGG-NH_2_-conjugated anti-EpCAM treated with MCF7 cells in the (ZGG-EpCAM) culture dish, and just ZGG-NH_2_ treated with MCF7 cells in the (ZGG-NH_2_) culture dish; two culture dishes were simultaneously exposure to UV light 2 min before collecting images; the interval time is 5 min between two pictures; **b** The ratio of persistent luminescence intensity from cells of (ZGG-EpCAM) culture dish divides by the persistent luminescence intensity from (ZGG-NH_2_) culture dish

To further support the recognition ability of ZGG-EpCAM nanoprobes to breast cancer cells, the persistent luminescence properties were also employed to probe recognition capacity of ZGG-NH_2_ modified or unmodified anti-EpCAM to MCF7 cancer cells. The IVIS Lumina II imaging system was used to quantify their luminescence change within 20 min from the different treated cells, and the luminescence intensity from treated cells with ZGG-EpCAM was stronger than that of treated cells with ZGG-NH_2_. We could detect the luminescence signals of both samples even after 20 min as shown in Fig. 7a. The luminescence intensity of the cells treated with ZGG-EpCAM nanoprobes was nearly 2.3 times higher than that of cells treated with ZGG-NH_2_ as shown in Fig. 7b, which might further reveal that the ZGG-EpCAM nanoprobes possessed relatively strong specific binding capability to breast cancer cells. Therefore, with the help of IVIS Lumina II imaging system, ZGG-EpCAM nanoprobes could be employed to realize quantitative luminescence signal detection of labeled breast cancer.

## Conclusion

In summary, we synthesized NIR emitting long-persistence luminescent ZGG nanoparticles by employing a sol–gel method. In order to enhance their specific capability to breast cancer cells, ZGG nanoparticles were modified with anti-EpCAM antibody to obtain ZGG-EpCAM nanoprobes. These nanoprobes not only possess a narrow-band emission peaked at 698 nm under excitation at 254 nm with excellent NIR long afterglow properties and low cytotoxicity but also possess targeting capability toward breast cancer cells (MCF7) which overexpressed EpCAM antigen. Further results of in vitro imaging confirm that ZGG-EpCAM possesses specific targeting capability toward breast cancer cells. More important, we could utilize ZGG-EpCAM long afterglow nanoprobes to realize the real-time monitored breast cancer with the help of IVIS Lumina II imaging system. This will improve the efficiency of detection in vivo at an early stage. We expect this study to provide a new perspective in the diagnosis of cancer.
